# Retraction: Ectopic Pregnancy-Derived Human Trophoblastic Stem Cells Regenerate Dopaminergic Nigrostriatal Pathway to Treat Parkinsonian Rats

**DOI:** 10.1371/journal.pone.0247151

**Published:** 2021-02-11

**Authors:** The PLOS ONE Editors

After this article [1] was published, the authors notified the journal that data were duplicated in Fig 4G and 4J; the aspect ratio differs for the corresponding blot images in the two panels. The authors offered an updated figure with replacement data in Fig 4J but commented that the raw data underlying Fig 4 are no longer available.

In following up on this issue, it came to light that the handling Academic Editor and two of the article’s authors are affiliated with the same institution (National Cheng Kung University). We sincerely regret that this potential competing interest was not identified and addressed prior to the article’s publication. In light of this issue, *PLOS ONE* reassessed the article with support from a different member of *PLOS ONE*’s Editorial Board and an external reviewer.

The following additional concerns were raised in the reassessment:

- The immunolabeling results reported in Fig 3C are of poor quality and do not provide clear evidence to support the claims made. The authors provided the original image and a revised figure but these did not resolve the concerns.
- The control and experimental panels in Fig 5C appear substantially different. The authors clarified that these data were obtained in different experiments conducted in different laboratories. As such, the findings reported in this figure and the related results statements are in question. The authors provided an alternative image but even considering this replacement data, the corresponding results statement in the article was not supported.
- The statistical methods used in the study were not appropriate and/or adequate in some cases, some of the statistical tests needed to support the findings were not conducted or were not reported, and the sample size was too low in some experiments to rigorously address the indicated questions. The authors offered to address the concerns about the reported statistics by providing results of reanalyses, but some of the original data are no longer available, and in light of the sample size issue, statistical reanalyses would not fully resolve these data concerns.
- The article did not include adequate controls for the immunohistochemistry experiments, many of the histological results were wrongly interpreted, and overall, the article did not report experimental evidence to support several of the article’s claims. For example, the following claims were not supported by the reported results:
  ○ “abundant newly generated TH-positive neurons appeared in the lesioned substantia nigra pars compacta (SNC) with multiple outgrowths to form neural circuitries with the surrounding host tissues”; and
  ○ the central conclusion that transplantation of trophoblastic stem cells regenerated the dopaminergic nigrostriatal pathway in a chronic Parkinson’s Disease rat model.
- There are multiple instances in which experiments and/or findings were not clearly or accurately reported in the article.

The authors offered replacement figures and text revisions to address the above issues. However, the *PLOS ONE* Editors determined that the concerns call into question the overall reliability of the results and conclusions reported in the published article, and so we retract this article.

TTYL, CFT, THH, JJJC, YCW, MCK, SS, EMT, and JNL did not agree with retraction and stand by the article’s findings. RMW either could not be reached or did not respond directly.

Note, the following information was omitted from the article’s [1] Competing Interests statement:

Authors TT-YL, E-MT, and J-NL are listed as inventors on patent application titled “Generation of neural stem cells from human trophoblast stem cells” (International Publication Number WO 2012/068170 A2, Application Number PCT/US201 1/060868; filed November 2011).
